# Fetal Fibroblasts and Keratinocytes with Immunosuppressive Properties for Allogeneic Cell-Based Wound Therapy

**DOI:** 10.1371/journal.pone.0070408

**Published:** 2013-07-24

**Authors:** Thomas Zuliani, Soraya Saiagh, Anne-Chantal Knol, Julie Esbelin, Brigitte Dréno

**Affiliations:** 1 Cell and Gene Therapy Unit, CIC Biotherapy INSERM 0503, Hôtel-Dieu University Hospital, Nantes, France; 2 CRCNA, INSERM U892 CNRS6299, Laboratory of Immuno-Dermatology, Hôtel-Dieu University Hospital, Nantes, France; 3 Gynecology and Obstetric Department, HME Hospital, Nantes, France; 4 Unit of Dermato-Cancerology, CIC Biotherapy INSERM 0503, Hôtel-Dieu University Hospital, Nantes, France; University of Birmingham, United Kingdom

## Abstract

Fetal skin heals rapidly without scar formation early in gestation, conferring to fetal skin cells a high and unique potential for tissue regeneration and scar management. In this study, we investigated the possibility of using fetal fibroblasts and keratinocytes to stimulate wound repair and regeneration for further allogeneic cell-based therapy development. From a single fetal skin sample, two clinical batches of keratinocytes and fibroblasts were manufactured and characterized. Tolerogenic properties of the fetal cells were investigated by allogeneic PBMC proliferation tests. In addition, the potential advantage of fibroblasts/keratinocytes co-application for wound healing stimulation has been examined in co-culture experiments with *in vitro* scratch assays and a multiplex cytokines array system. Based on keratin 14 and prolyl-4-hydroxylase expression analyses, purity of both clinical batches was found to be above 98% and neither melanocytes nor Langerhans cells could be detected. Both cell types demonstrated strong immunosuppressive properties as shown by the dramatic decrease in allogeneic PBMC proliferation when co-cultured with fibroblasts and/or keratinocytes. We further showed that the indoleamine 2,3 dioxygenase (IDO) activity is required for the immunoregulatory activity of fetal skin cells. Co-cultures experiments have also revealed that fibroblasts-keratinocytes interactions strongly enhanced fetal cells secretion of HGF, GM-CSF, IL-8 and to a lesser extent VEGF-A. Accordingly, in the *in vitro* scratch assays the fetal fibroblasts and keratinocytes co-culture accelerated the scratch closure compared to fibroblast or keratinocyte mono-cultures. In conclusion, our data suggest that the combination of fetal keratinocytes and fibroblasts could be of particular interest for the development of a new allogeneic skin substitute with immunomodulatory activity, acting as a reservoir for wound healing growth factors.

## Introduction

Cell-based engineered skin substitutes are promising to treat difficult-to-heal acute and chronic wounds such as large/deep burns, ulcers resistant to conventional therapies or surgical wounds [1]–[5]. Cultured autologous epidermal cell-based therapy is used for more than two decades as permanent wound coverage for large burns [6]. Although this technique has been shown to improve outcomes in patients with large burn injuries, its clinical use is limited by the creation of a second wound at the donor site, the three-week delay needed to obtain sufficient amounts of cells, and the absence of a dermal component resulting in low graft take and wound contraction. Concurrently, allogeneic cell-based engineered skin substitutes have been developed. Where they offer off-the-shelf temporary wound coverage acting as biologically active dressings releasing growth factors, cytokines and extra cellular matrix (ECM) components essential for proper wound healing, they are susceptible of immune rejection [7], [8]. Among these skin substitutes, bilayered constructs associating neonatal foreskin epidermal and dermal cell layers are the most developed. Two of them are currently marketed (Apligraf, Organogenesis Inc., Canton, MA, USA; OrCel, Ortec International Inc., New York, NY, USA) and have been shown to promote healing in chronic non-healing venous ulcers and of burn patient donor site wounds [9], [10].

Because of their low immunogenicity, and their wound healing properties, fetal skin cells represent an attractive alternative to the commonly used neonatal foreskin keratinocyte and fibroblast cell-based engineered skin substitutes. Fetal skin, before the third trimester of gestational age, heals rapidly without scar formation conversely to adult skin. Minimal inflammation, specific cytokine and growth factor profiles, and faster and organized deposit and turnover of ECM components during fetal wound healing have been proposed to explain the absence of scar formation [11]–[13]. Interestingly, this phenomenon appears to be largely dependent on the fetal tissue itself and not rely on the specific *in utero* environment [14], [15], conferring great intrinsic potential to fetal skin cells for wound healing management. This has been investigated in two phase I clinical trials for the treatment of pediatric burns [16] and resistant leg ulcers [17], providing first evidences of the therapeutic benefit of fetal fibroblasts for the treatment of acute or chronic skin wounds.

This study was conducted in order to further develop an allogeneic fetal cell-based dressing for acute and chronic wound management. Considering that keratinocyte-fibroblast interactions play a critical role in the wound healing process, we hypothesized that fetal cell-based therapy for cutaneous wounds could be improved by combining fetal fibroblasts and keratinocytes. As no method describing how to produce sufficient amounts of fetal keratinocytes that would be needed for future cell therapy development was found in the literature, we developed a specific method to isolate, amplify and bank clinical grade keratinocytes and fibroblasts from a single fetal skin sample. Then, to test the relevance of using these cells for further development of an allogeneic fetal cell-based skin substitute, fibroblasts and keratinocytes from both clinical batches were first characterized. Then, their immunogenicity and immunosuppressive properties were examined to test their potential for allotransplantation. Finally, advantage of fibroblasts/keratinocytes co-application for wound healing stimulation has been examined by co-culture experiments.

## Materials and Methods

### Fetal Skin Samples

The approval of the French agency “Agence de la Biomédecine” was obtained for all procedures and the informed consent document (registered n° PFS08-016). All patients included in this research program were fully informed by the clinicians and signed the approved written informed consent form. Tissue samples (n = 7) were obtained from patients undergoing medically induced abortions. Human fetal skin was obtained from the back of 7 gestational age fetuses of 18–24 weeks without chromosomal abnormalities and viral infections by HBV, HCV, HIV I and II, HTLV I and II, and CMV. Following surgical procedures, skin specimens were immediately immersed in transport medium consisting of DMEM glutamax (Invitrogen, #31966-047, Cergy-Pontoise, France)/UV-irradiated FBS (20%) (Invitrogen, #10094142)/Penicillin-Streptomycin (2%) (Lonza, #DE17-603E, Verviers, Belgium), and transferred to the Cell Therapy Unit of Nantes University Hospital within one hour.

### Fetal Fibroblast and Keratinocyte Banking Process

In this study, all the analyses were performed on fibroblasts and keratinocytes from two cell banks that were manufactured from a single skin specimen from a 19-week gestational age fetus, as described below (See supporting information, Figure S1 for schematic representation of the manufacturing process). Fetal skin was rinsed twice in DPBS (Lonza, #BE17-512F) containing penicillin-streptomycin (2%) and divided into explants (<0.5 cm^3^). Ten skin explants were plated per well of 6-well plates and incubated in DMEM glutamax (Invitrogen)/irradiated FBS (20%)/penicillin-streptomycin (5000 UI/ml) (1%) at 37°C in moist atmosphere with 5% CO_2_. Culture medium was renewed every 3–4 days. First cells to appear were the keratinocytes (as early as 2 days after plating), forming a ring around the explants. Rapidly, the fibroblasts also grew out of the explants until the wells were coated by fibroblasts. After 17 days, fibroblasts reached confluence. At this stage, fibroblasts and keratinocytes were differentially dissociated using trypsin-EDTA for 3 to 5 minutes in order to detach the fibroblasts but not the keratinocytes. Fibroblasts were transferred into a 50 ml tube, and subcultured in flasks at the density of 8000 cells/cm^2^ (passage 1). The remaining adherent keratinocytes were gently rinsed and covered with 2 ml of keratinocyte growth medium CnT-07 (CellnTec, # CnT-07, Bern Switzerland). After 7 additional days, keratinocytes reached confluence and were trypsinized, centrifuged, counted and plated in flasks at the density of 4000 cells/cm^2^ (passage 1). At the end of passage 1, two initial cell banks (ICB) of fetal fibroblasts and keratinocytes were constituted. Then, starting from one vial of each ICB, cells were thawed, amplified for two additional passages prior freezing to constitute two clinical batches of fetal fibroblasts and keratinocytes at passage 3. Fibroblasts and keratinocytes were frozen at the density of 1×10^6^ cells/ml in each vial in human albumin (4%) (LFB, Lille, France)/DMSO (10%) (B.Braun, #2791102, Boulogne, France) by lowering the temperature of 1°C/min until a temperature of −80°C was reached and then stored in vapor nitrogen container.

### Molecular Characterization Techniques

#### Flow Cytometry

For flow cytometry, 0.2×10^6^ cells were stained along with appropriate isotypic controls. Briefly, cells were rinsed twice in PBS, stained with anti-ß1 integrin-FITC mAb, anti-α2 integrin-FITC mAb, anti-α3 integrin-FITC mAb (Tebu-bio, respectively #SC-18887FITC, #SC-53352 FITC and #SC-32237FITC, Le-Perray-en-Yvelines, France, dilution 1/100) or anti-CD1a-FITC mAb (Tebu-bio, #SC-5265-FITC, dilution 1/200) in 200 µL of DPBS/BSA (0.05%) (Sigma-Aldrich, #A9418, Saint-Quentin Fallavier, France) for 30 minutes at 4°C protected from light. For MHC class I and II stainings, cells were stained with HLA-I primary antibody recognizing HLA-A, -B, -C or HLA-II primary antibody recognizing HLA-DP, -DQ, -DR (BD Biosciences, respectively #555551 and #555557, Le Pont de Claix, France, dilution 1/200) followed by goat anti-mouse PE-alexa680 conjugated secondary antibody (Invitrogen, #A-20983, dilution 1/200). Then, cells were washed twice in DPBS and resuspended in DPBS/PFA (1%) until flow cytometry analysis. For K14, P4H and HMB45 intracellular staining, 0.25×10^6^ cells were rinsed twice in DPBS, permeabilized using fixation/permeabilization buffer set (eBiosciences, #00-8333-56, Paris, France) according to the manufacturer’s instructions and stained with rabbit anti-K14 (Abcam, #ab15461, Paris, France, dilution 1/500), mouse anti-P4H (Abcam, #ab44971, dilution 1/100) or anti-HMB45 (Tebu, #sc-59305, 1/100) Abs for 1 hour, along with the appropriate isotypic controls. Cells were rinsed twice and then stained with goat anti-mouse Alexa 488 or goat anti-rabbit Alexa 488 secondary antibodies (Invitrogen, respectively #A-11029 and #A-11034, dilution 1/500 in permeabilization buffer). A minimum of 10^4^ viable cell gated events were acquired on a FACScalibur flow cytometer using Cell Quest software (Becton Dickinson, Grenoble, France) and data were analyzed using WinMDI software (developed by JC Trotter).

#### Immunofluorescence

For immunofluorescence, fetal keratinocytes and fibroblasts from both clinical batches were seeded at the density of 30,000 cells/cm^2^ in chamber slides (Labtek, Fischer scientific, Illkirch, France), incubated overnight at 37°C in moist atmosphere with 5% CO_2_. Cells were rinsed with DPBS, fixed/permeabilized in ice cold methanol for 2 minutes, rinsed twice more, saturated with DPBS/FBS (10%) for 30 minutes, and incubated simultaneously with mouse anti-P4H (1/100) and rabbit anti-K14 (1/1000) Abs in DPBS/10% FBS for 2 hours at room temperature. Then, cells were rinsed twice in DPBS and incubated with secondary goat anti-mouse Alexa488 (1/500) and anti-rabbit Alexa594 (Invitrogen, #A-11012, 1/500) Abs in DPBS/10% FBS for 1 hour and finally counterstained with DAPI (Invitrogen, #D3571) for 10 minutes at the final concentration of 1 µg/ml in DPBS. Finally cells were rinsed twice in DPBS and slides were mounted before analysis using a Zeiss Axiovert 200-M inverted microscope (Carl Zeiss, Le Pecq, France) and the AxioVision 4.6 program.

#### Immunohistochemistry

For immunohistochemistry, fetal skin fragments were embedded in OCT compound (Gentaur, #4583-01, Paris, France) and 5-µm thick sections were cut, mounted on superfrost+slides, fixed in acetone for 10 minutes, dried for an additional 10 minutes and stored at −20°C until immunostaining. Frozen sections were stained by standard immunoperoxidase techniques using ABC kit (DAKO, #K5003, Trappes, France) according to the manufacturer’s procedure with primary antibodies mouse anti-IDO-1 (1/100, Abd Serotec, #OBT2037G, Colmar, France) and goat anti-IDO-2 (1/200, Novus biological, #NBP1-45209, Cambridge, UK) or isotypic controls. For IDO-2 immunodetection, the secondary biotinylated rabbit anti-goat was used (DAKO, #E0466). Slides were counterstained with Mayer’s haemalum before mounting using aqueous medium (Diagnostic biosystems, #K002, Pleasanton, USA). Slides were read by microscopy (Leitz microscope) at ×400 magnification and photographed using a digital camera (Nikon, D70S).

### Preparation of Cell Culture Supernatants and Multiplex Cytokine Assay

A multiplex cytokine assay was performed to compare cytokine release from fetal and adult cells and to study fibroblasts-keratinocytes interactions. For adult fibroblasts and keratinocytes preparation, adult skin (n = 3) were obtained from adult caucasian patients undergoing plastic surgery. Passage three fetal and adult fibroblasts and keratinocytes were thawed and incubated at 37°C in 5% CO_2_ for 72 hours for cell recovery. For each sample, a total of 350 000 cells (fibroblasts, keratinocytes or a mix, at the ratio 1∶1, of 175 000 fibroblasts and 175 000 keratinocytes) per well of a 6-well plate were seeded in 3 ml of culture medium. After 48 hours of incubation, supernatants were recovered, centrifuged and stored at −80°C. A multiplex immunoassay based on Luminex technology was performed in duplicate according to the manufacturer’s guidelines using the Procarta Immunoassay kit (ebiosciences), to reveal PDGF-BB, VEGF-A, bFGF, IL-1α, IL-8, IL-10, GM-CSF, HGF and TGF-β1 in cell culture supernatants.

### In vitro Scratch Assay

Fibroblasts, keratinocytes or both cell types in co-culture at the ratio 1∶1 were seeded in 12-well plates at the density of 1×10^5^ cells/well. Plates were maintained at 37°C and 5% CO_2_ in moist atmosphere overnight in order to obtain a confluent cell monolayer. The confluent monolayers were scratched with pipette tip and culture medium was immediately removed and replaced by non-supplemented fresh medium. Time-lapse video-microscopy experiments were performed using a Leica DMI 6000B. Plates were placed inside an incubator chamber that maintained the environmental CO_2_ concentration at 5% for the duration of filming. Digital pictures of four areas/scratch were acquired using MetaMorph v.7.5 software (MDS, Foster City, CA) and saved every 10 min over 20 h. The digitised images were then analysed using image-J software to measure the percentage of wound area coverage. Data are presented as the extent of wound closure at T0, T5 and T20 hours.

### Allogeneic PBMC Proliferation Assay

Proliferation assays of PBMC from healthy donors in the presence of fetal fibroblasts, keratinocytes or both in co-culture (ratio 1∶1) were performed in 96-well plates. Forty-eight hours after thawing, fetal cells were irradiated (65 Gy), seeded (in triplicate) at 30,000, 6000, 3000 and 600 cells/well and allowed to attach overnight at 37°C in 150 µl RPMI 1640 (Lonza, #12.702 F) supplemented with 10% FBS. Total PBMC were isolated from 50 ml human blood samples from normal individuals by density gradient sedimentation with lymphocyte separation medium (Eurobio, #CMSMSL01-0U, Les Ulis, France). Isolated PBMC were rinsed twice in RPMI 1640 and 120,000 cells in 50 µl RPMI 1640 supplemented with 10% FBS with or without 600 UI/ml IL-2 (Novartis, Proleukin®) and 4 µg/ml PHA-L (Sigma-Aldrich, #L4144) were added to fetal irradiated cells. To evaluate the role of IDO activity, 1-Methyl-D-Tryptophan (1-MT) (Sigma-Aldrich, #447439) was added with PBMC at a final concentration of 2, 20 and 200 µM. Plates were incubated at 37°C for 72 h, then pulsed for additional 16 h with 1 µCi/well [^3^H]-thymidine (Perkin Elmer, #NET027L001MC, Courtaboeuf, France), before cell harvesting and counting in a ß-plate scintillation counter (Perkin Elmer, GE Healthcare, Orsay France).

### Statistical Analysis

Values are presented as mean ± SD. The student’s unpaired two-tailed t-test was used to determine significant differences between samples for scratch-wound assays and for PBMC proliferation assays. *P*-values less than 0.05 were considered statistically significant.

## Results

### Fetal Fibroblast and Keratinocyte Clinical Batch Manufacturing and Characterization

#### Fetal fibroblasts

**Figure 1:**
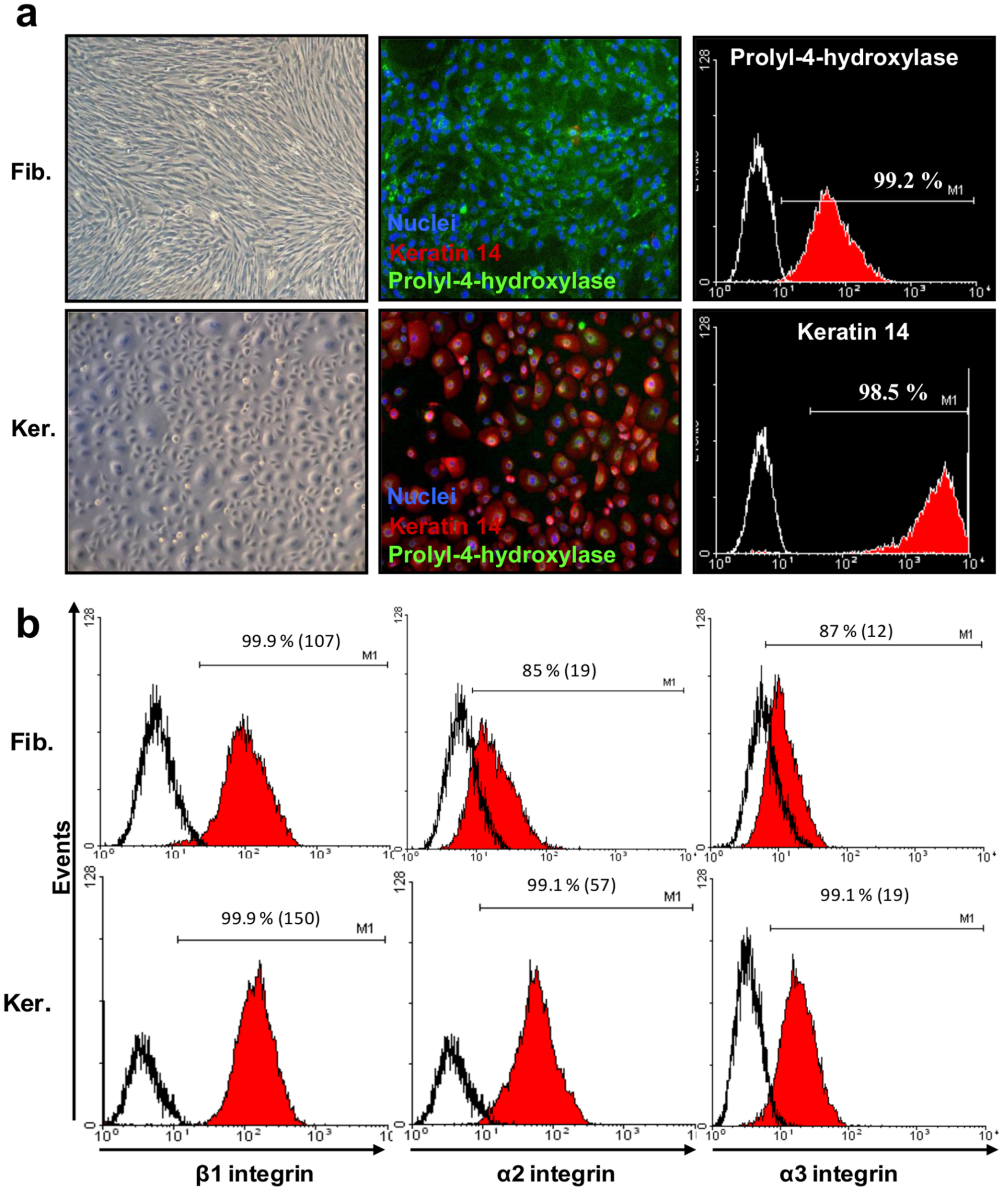
Characterization of the fetal fibroblasts and keratinocytes manufactured of the clinical batches. **A**, Morphological and molecular characterization. Left panel, microphotography of the fetal fibroblasts and keratinocytes. Middle panel, immunofluorescence of cultured fetal fibroblasts and kerationcytes from the clinical batches, stained for nuclei (blue), prolyl-4-hydroxylase (green) and keratin 14 (red). Right panel, cytometry histograms showing the percentage of prolyl-4-hydroxylase positive cells and the percentage of keratin14 positive cells in the fetal fibroblasts and keratinocytes, respectively. Full histograms represent test samples, solid lines represent isotypic controls. **B**, Integrin expression profiles. Cytometry histograms showing β1-, α2- and α3-integrin expression in fetal fibroblasts (upper panel) and keratinocytes (lower panel). Cytograms are typical of at least three independent experiments.

At the end of the explant outgrowth (P0), a total of 1.45×10^6^ fibroblasts were obtained. They were further amplified for one additional passage and frozen to create an initial cell bank (ICB) of 16 vials (10^6^ cells/vial). Then, from 1 ICB vial, after two additional passages, a clinical batch of 230 vials of fetal fibroblasts (1×10^6^ cells/vial, cell viability 96%) was constituted. Hence, potentially 3.68×10^9^ fibroblasts could be obtained. All cells showed a fibroblast-like morphology (Figure 1A, upper left panel). This was supported by immunofluorescence data showing that after triple staining of P4H, K14 and nuclei (Figure 1A, upper middle panel), the fetal fibroblasts expressed P4H but not K14 and confirmed by flow cytometry analysis demonstrating that 99.2% of cells expressed the prolyl-4-hydroxylase, a marker of fibroblasts (Figure 1A, upper right panel). Further analysis using flow cytometry indicated that fetal fibroblasts highly expressed the β_1_-integrin on their surface membrane (100% of positive cells, MFI = 107) (figure 1B) whereas α_2_- and α_3_-integrins were weakly expressed (respectively, 85% of positive cells, MFI = 19 and 87% of positive cells, MFI = 12) (figure 1B).

#### Fetal keratinocytes

At P0, after fibroblast removal, the remaining keratinocytes were amplified in CnT-07 growth medium until they reached 70–80% confluence. At the end of the explant outgrowth, a total of 1.18×10^6^ keratinocytes were recovered. As for fibroblasts, keratinocytes were further amplified in flasks for one additional passage and frozen to create an ICB of 13 vials (1×10^6^ cells/vial). Then, from 1 ICB vial, after two additional passages, a clinical batch of 106 vials of fetal keratinocytes (1×10^6^ cells/vial, cell viability 98%) was constituted. Hence, potentially 1.272×10^9^ keratinocytes could be obtained. Cells showed a keratinocyte-like morphology (Figure 1A, lower left panel). This was supported by immunofluorescence showing that the fetal keratinocytes highly expressed K14 (Figure 1A, lower middle panel) and confirmed by flow cytometry analysis demonstrating that 98.5% of cells were positive for K14 (Figure 1A, lower right panel). Further analysis by flow cytometry indicated that fetal keratinocytes expressed β_1_-, α_2_- and α_3_-integrins on their surface membrane (respectively 99.9% of positive cells, MFI = 150, 99.1% of positive cells, MFI = 57, and 99.1% of positive cells, MFI = 19) (figure 1B).

In both fibroblast and keratinocyte clinical batches, CD1a positive cells (Langerhans cells) and HMB45 positive cells (melanocytes) were not detected (Supplementing information, Figure S2).

### MHC Class I and II Expression in Fetal Fibroblasts and Keratinocytes

**Figure 2:**
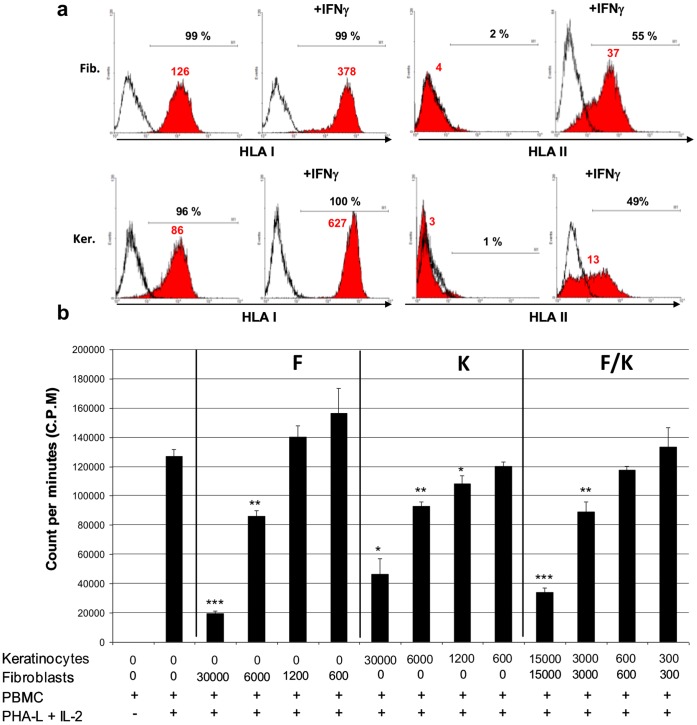
Immunogenicity and Immunosuppressive activity of fetal cells. **A,** MHC class I and class II expression in fetal cells. Flow cytometry analysis of MHC class I and class II expression in fetal fibroblasts and kerationcytes treated or not with 1 µg/ml IFN-γ for 48 hours. Full histograms represent test samples, solid lines represent isotypic controls. Percentage of positive cells and mean fluorescence intensity values (MFI) are indicated. Cytograms are typical of two independent experiments. **B,** Proliferation of allogeneic PBMC in co-culture with fetal fibroblasts, keratinocytes or both cell types in co-culture. PBMC were initially plated with various fetal cell numbers corresponding to four different ratios between fetal cells and PMBC, 1∶4, 1∶20, 1∶100 and 1∶200. PBMC were collected from the co-cultures after 5 days and treated with ^3^H-thymidine for 16 hours before radioactive counting. Data represent the means ± SD (n = 3) of ^3^H-thymidine incorporation by proliferative PBMC. Asterisks denote significant differences in PBMC proliferation compared to the positive control (column 2) (*, p<0.05; **, p<0.01; ***, p<0.001). Data are representative of three independent experiments performed with PBMC obtained from different healthy donors.

We observed by flow cytometry that both fetal fibroblasts and keratinocytes did not express MHC class II but expressed MHC class I (HLA -A, -B and -C) (Figure 2A). When stimulated with 500 UI/ml IFN-γ for 48 hours, MHC class I and II expression was increased in both cell types (Figure 2A). A 3-fold increase in the MFI values was observed for MHC class I and more than 9-fold increase for MHC class II in fetal fibroblasts while a 7-fold and 4.3-fold increase was observed respectively for MHC class I and class II in fetal keratinocytes (Figure 2A).

### Inhibition of PBMC Proliferation in Co-culture with Fetal Fibroblasts or Keratinocytes alone and both Cell Types in Co-culture

To further investigate the possibility of using fetal cells for allogeneic transplantation, we performed an *in vitro* lymphoproliferation assay. We found that both fibroblasts and keratinocytes dramatically inhibited the proliferation of immune cells (PBMC) stimulated with IL-2 and PHA-L. As shown in figure 2B, this effect was dose-dependent and the maximum lymphoproliferation inhibition was obtained when 0.3×10^5^ fetal fibroblasts were co-cultured with 1.2×10^5^ stimulated PBMC. At this 1∶4 ratio, fetal fibroblasts inhibited lymphocyte proliferation by 85%, fetal keratinocytes by 64% and fibroblasts and keratinocytes in co-culture by 73%. This inhibitory effect on lymphocyte proliferation disappeared when fetal cells were co-cultured with lymphocytes at the ratio 1∶100 or 1∶200 (Figure 2B).

### IDO Activity is Responsible for the Immunosuppressive Properties of Fetal Fibroblasts and Keratinocytes

**Figure 3:**
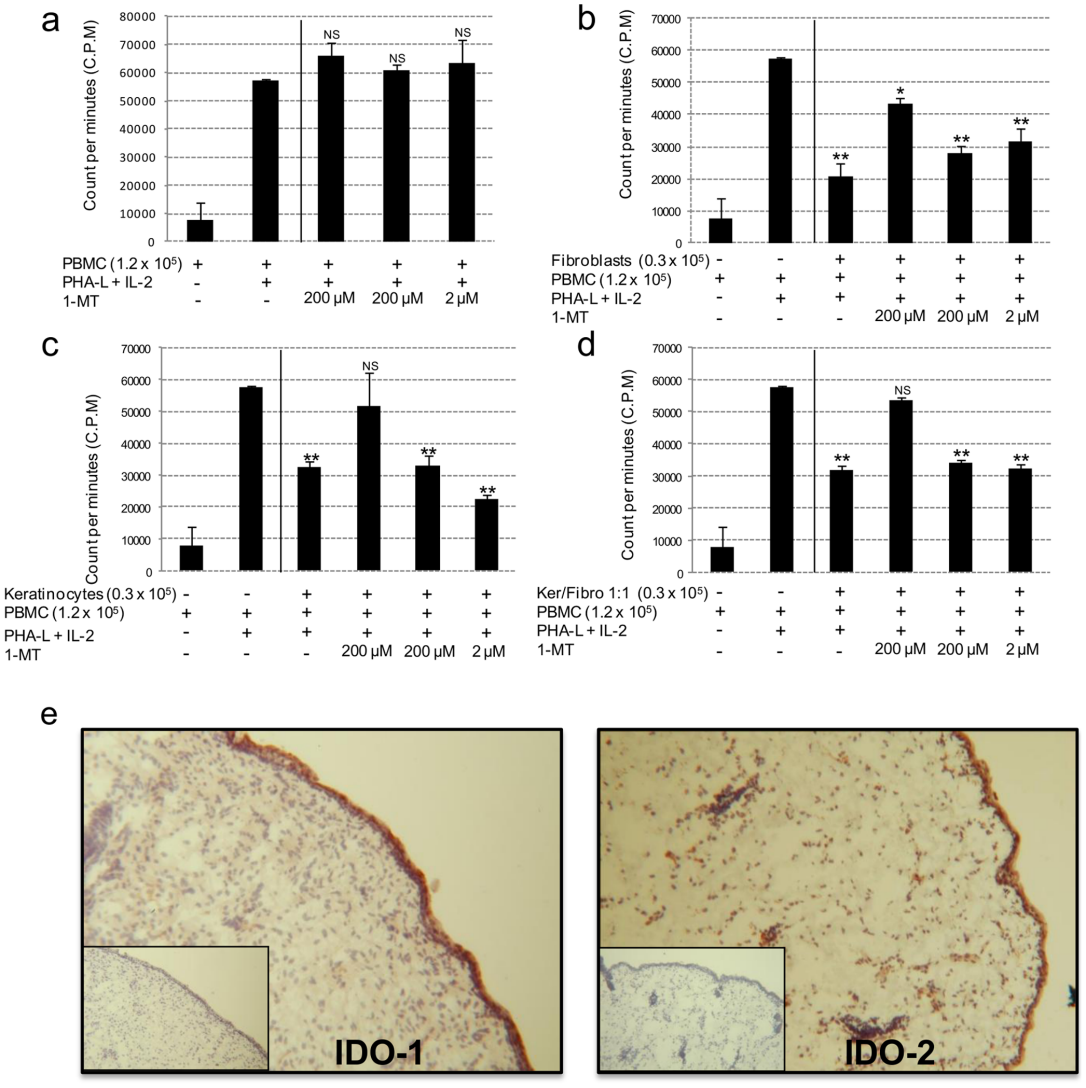
Indoleamine 2,3-dioxygenase (IDO) activity is responsible for the immunosuppressive activity of fetal cells. **A**, Effect of 1-MT on the proliferation of allogeneic PBMC co-cultured with fetal fibroblasts, keratinocytes or both cell types in co-culture. PBMC were initially plated with fetal cells at the ratio 1∶4. 1-MT was added at various concentrations to inhibit IDO activity. PBMC were collected from the cultures after 5 days and treated with 3H-thymidine for 16 hours before radioactive counting. Data represent the means (n = 3) ± SD of ^3^H-thymidine incorporation by proliferative PBMC. Asterisks denote significant differences in PBMC proliferation compared to the positive control (column 2) (*, p<0.05; **, p<0.01). Data are representative of three independent experiments performed with PBMC obtained from different healthy donors. **B**, IDO-1 and IDO-2 expression in fetal skin. Pieces of the skin sample used to manufacture the fibroblast and keratinocyte cell banks were frozen and embedded for cryosectioning. Skin sections were stained for the immunodetection of IDO-1 (left panel) and IDO-2 (right panel). Isotypic controls for IDO-1 and IDO-2 staining are indicated.

Indoleamine 2,3 dioxygenase (IDO) is a rate-limiting enzyme of the tryptophan catabolism. By its ability to locally decrease the tryptophan availability, IDO is recognized to exert an immunomodulatory effect on T-cells requiring tryptophan to proliferate. To test whether IDO could be responsible for the fibroblast- and keratinocyte-induced inhibition in lymphocyte proliferation, we used the inhibitor of IDO, 1-MT (1-methyl-D-tryptophan), to evaluate its impact on PBMC proliferation in co-culture with fetal cells. In a first set of experiments, 1-MT by itself had no effect on PBMC proliferation whatever the concentrations tested (Figure 3A). Figure 3B, C and D show that 1-MT abolished dose-dependently the inhibition in PBMC proliferation induced by fetal fibroblasts or keratinocytes alone and both in co-culture.

### IDO-1 and IDO-2 are Expressed by Fetal Skin Cells

We next investigated IDO-1 and IDO-2 expression in the fetal skin that was used to manufacture the fibroblast and keratinocyte clinical batches. As shown in Figure 3E, we found that IDO-1 and IDO-2 were expressed in fetal epidermal and dermal skin compartments. IDO-1 was preferentially expressed in the epidermal compartment whereas IDO-2 was expressed both in the dermal and epidermal compartments.

### Cytokines/Wound Healing Growth Factors Released by Fetal and Adult Keratinocytes and Fibroblasts

**Figure 4:**
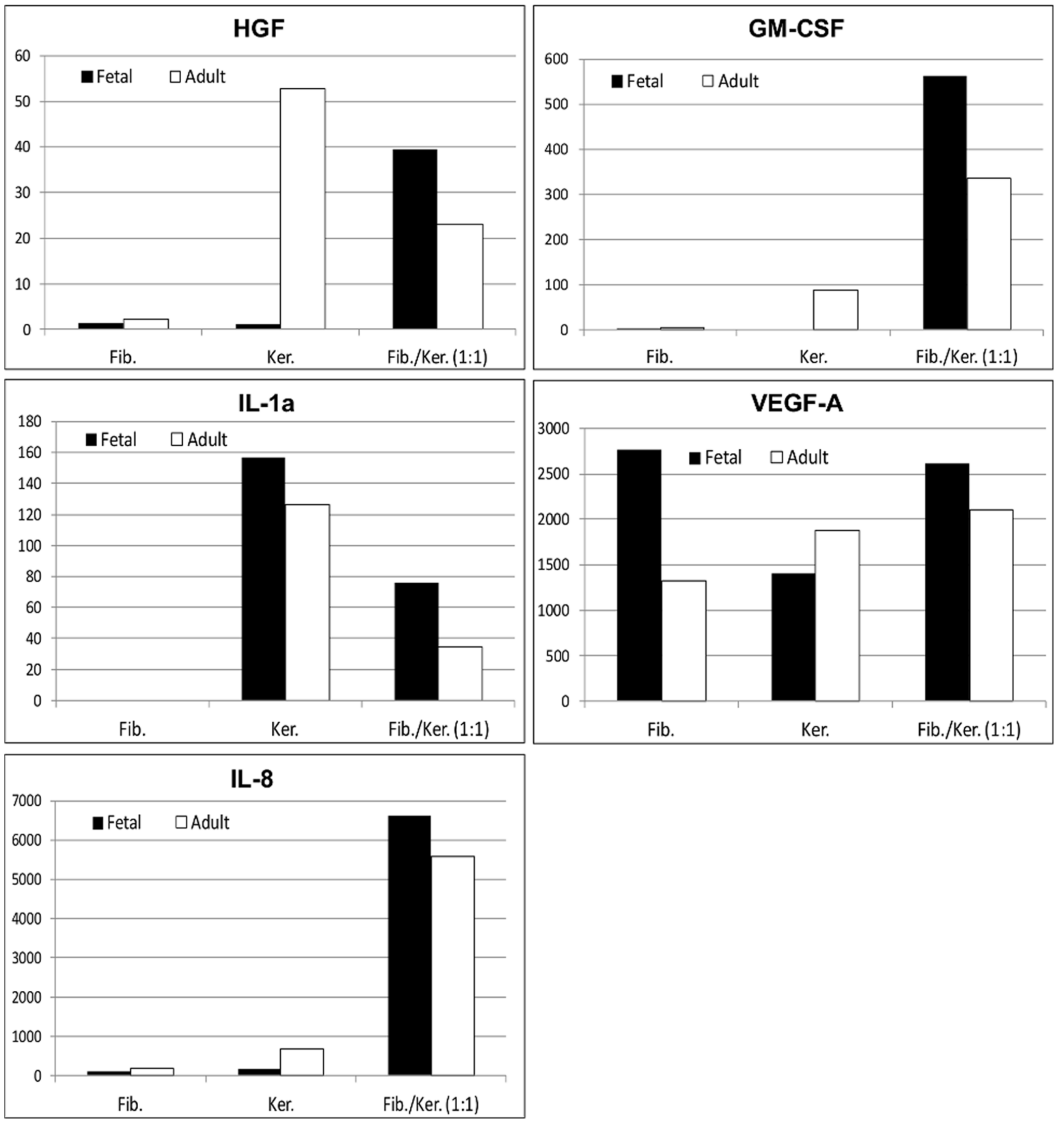
Multiplex cytokine analysis of cell culture supernatants from fetal and adult fibroblasts and keratinocytes mono-cultures or fibroblasts and keratinocytes co-cultures. Supernatants from fibroblast and keratinocyte mono-cultures or both cell types in co-culture (ratio 1∶1) were collected to detect soluble HGF, GM-CSF, IL-1α, VEGF-A and IL-8 by multiplex immunoassay (see materials and methods). For adult skin cells, values are the means of three samples (see table 1). Fib., fibroblasts mono-culture; Ker., keratinocytes mono-culture; Fib./Ker. (1∶1), fibroblasts and keratinocytes co-culture at the ratio 1∶1.

Cytokines/wound healing growth factors release in supernatants of fetal and adult fibroblasts and keratinocytes mono-cultures and co-cultures were assessed by multiplex cytokine assay. Factors secretion by fetal cells was compared with those of adult fibroblasts and keratinocytes (Figure 4 and Table S1). Analysis revealed that fetal fibroblasts secreted higher amounts of VEGF-A (2770 pg/mL) than adult cells (1323 pg/mL). HGF, GM-CSF, IL-1α and IL-8 were found to be secreted by fibroblasts at a low level, without significant differences between fetal and adult cells. Fetal keratinocytes secreted higher amounts of IL-1α compared to adult keratinocytes (157 vs 126 pg/ml) but lower level of HGF (53 vs 1 pg/ml), GM-CSF (87 pg/ml vs non detectable level), VEGF-A (1875 vs 1400 pg/mL) and IL-8 (670 vs 165 pg/mL). Finally, cytokines release by co-cultured fibroblasts and keratinocytes was found to be higher with fetal cells than adult for HGF (39 vs 23 pg/mL), GM-CSF (562 vs 334 pg/ml) IL-1α (76 vs 34 pg/ml), VEGF-A (2617 vs 2337 pg/ml) and IL-8 (6331 vs 5590 pg/ml). PDGF-BB, TGF-β1, IL-10 and bFGF were not detected in any cell culture supernatants (data not shown).

### Scratch Closure is Faster in Fetal Fibroblast and Keratinocyte Co-cultures than in Fibroblast or Keratinocyte Mono-cultures

**Figure 5:**
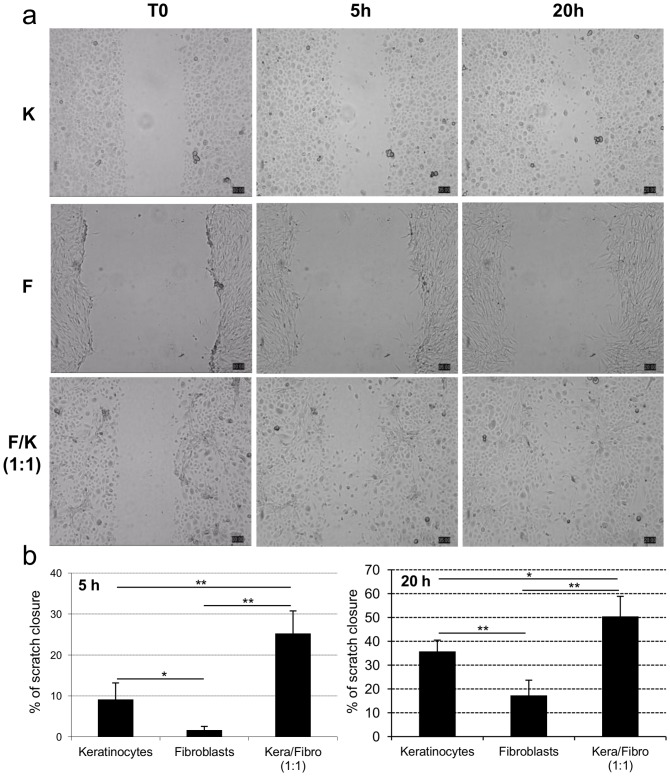
Scratch closure is faster in fetal fibroblast and keratinocyte co-cultures than in fibroblast or keratinocyte mono-cultures. **A**, Representative images of the progression of the scratch closure at T0, 5 h and 20 h for keratinocytes (upper panel), fibroblasts (middle panel) and both in co-culture (lower panel). **B**, The extent of the scratch closure in scratch assays performed on fetal fibroblasts, keratinocytes or both in co-culture at 5 h and 20 h. Data shown are the means ± SD (n = 4). Asterisks denote significant differences in the extent of the scratch closure (*, p<0.05; **, p<0.01).

Scratch assays were performed to evaluate the ability of fibroblasts and keratinocytes alone or in co-culture to close a wound *in vitro*. In scratch assays, the extent of the scratch closure was 1.7% (±0.9) and 9% (±4) for fibroblasts and keratinocytes, respectively (figure 5A) at T5 and 17% (±6) and 35% (±4) for fibroblasts and keratinocytes, respectively at T20 (figure 5A). In co-cultures, cells closed faster the scratch area, the extent of the scratch closure being 25% (±5) at T5 and 50% (±8) at T20 (Figure 5A and 5B). For further illustration of these results see supporting information, Video S1, Video S2 and Video S3 showing a scratch closure by fetal keratinocytes, fibroblasts and both cell types in co-culture.

## Discussion

Since the 70 s, increasing attention is given to fetal wound healing with the observations that fetal skin heals rapidly without scar formation [18]. Although the mechanisms underlying fetal regeneration have been widely studied, to date, the therapeutic potential of fetal skin cells has poorly been studied at the clinical level. Only recently, a skin substitute, composed of fetal fibroblasts associated to a collagen matrix, was shown to be promising for the treatment of both acute and chronic wounds [16], [17]. However, neither fetal epidermal cells nor the association of fetal dermal and epidermal cells have been studied. One of the reasons is the difficulty in isolating, culturing, amplifying and banking sufficient amounts of fetal epidermal cells for clinical use. Here, we successfully developed a method allowing manufacturing of two clinical grade batches of keratinocytes and fibroblasts from a single fetal skin sample. According to a recent paper [19], we found that the optimal approach to establish a keratinocyte culture from a fetal skin sample was the outgrowth method. Other methods including the conventional enzymatic digestion with dispase for dermis-epidermis dissociation, then trypsin to obtain an epidermal cell were ineffective. Main reason was the extreme sensitivity to trypsin of the freshly isolated fetal epidermal cells. In the particular manufacturing process that we developed here, the critical step was the differential trypsinization performed when keratinocytes formed a thin ring around the explants while fibroblasts reached confluence. However, we took advantage of the fact that keratinocytes in DMEM/FBS were extremely cohesive around the explants and highly adherent, making the specific fibroblast detachment by differential trypsinization relatively easy. Then, after fibroblast removal, the switch from DMEM/FBS to CnT-07 medium allowed us to largely amplify the remaining keratinocytes. By this way, we initiated two independent cell cultures of fetal fibroblasts and keratinocytes that were amplified until the third passage. While it was possible to further expand fetal cells for additional passages, the choice to freeze the clinical batches at a relatively early passage was based on integrin expression analysis. Indeed, integrins are essential for both keratinocytes and fibroblasts during the wound healing process since they regulate various physiological cellular processes such as adhesion, migration, proliferation and differentiation [20], [21]. Here, we observed that fetal keratinocytes highly expressed the β_1_-, α_2_-, and α_3_-integrins while fibroblasts expressed only but highly the β_1_-isoform in culture. As a decreased integrin expression could negatively impact on wound healing, one of our concerns was to maintain a stable integrin expression level between culture initiation and clinical batch freezing. Particularly for keratinocytes, the switch between proliferation and differentiation is accompanied by a decreased integrin expression and notably the β_1_-integrin [22], [23]. Because we had previously observed that β_1_- and α_2_-integrin expression decreased from passage 4, coinciding with a decreased proliferation rate (data not shown), we chose to freeze keratinocyte clinical batches at the third passage.

Compared to their adult counterpart, fetal cells present the biological advantage of having an immunological privilege, allowing the feto-maternal tolerance [24]. While allorejection is dependent upon recipient T lymphocytes responding to class I and II cell surface molecules encoded by HLA genes [16], we showed that both fetal derived skin fibroblasts and keratinocytes did not express MHC class II and low level of class I. Nonetheless, the upregulated expression of both MHC class I and II observed upon IFN-γ stimulation, suggests that alloreactivity of fetal fibroblasts and keratinocytes could be enhanced in chronic cutaneous ulcer and burn wounds, where IFN-γ contributes to the local inflammation [25], [26]. This result prompted us, to further evaluate the possibility of using fetal derived skin cells for allogeneic transplantation, by studying their immunosuppressive properties. In the late nineties, it has been demonstrated that feto-maternal tolerance is mainly due to the fetal allograft ability to actively defend itself from attack by maternal T cells [27]. This immunosuppressive activity has been shown to rely on IDO, the rate-limiting enzyme for tryptophan metabolism. By creating a tryptophan deficient microenvironment, an amino acid required for T-cell proliferation, IDO expression, in fetal-derived syncytiotrophoblasts at the feto-maternal interface, is responsible for the local immunosuppression. Mechanisms of IDO immunosuppression have been further elucidated [28]–[30] and IDO-mediated tryptophan catabolism was also identified as a novel T-cell inhibitory effector mechanism in antigen presenting cells [31] and mesenchymal stem cells [32]. In this study, we showed that fetal fibroblasts and keratinocytes can dramatically decrease the proliferation of stimulated allogeneic PBMC. We further demonstrated that IDO activity is responsible for this immunosuppressive function, since specific inhibition of IDO with the competitive inhibitor 1-MT, reversed the anti-proliferative activity of fetal cells on PBMC. Consistently, we found that both IDO isoforms, IDO-1 and IDO-2, were expressed in fetal skin in the dermal epidermal compartments. These data suggest that an allogeneic skin substitute composed of fetal fibroblasts and keratinocytes, with intrinsic immunosuppressive activity, could have a prolonged survival after grafting by locally creating a tolerogenic microenvironment. In accordance to this, in a rabbit ear model, Chavez-Munoz et al. [33], have demonstrated that a skin engineered bio-construct composed of keratinocytes and IDO-transduced foreskin fibroblasts, can survive for up to 35 days after × enotransplantation. This prolonged survival was attributed to IDO activity of the transduced fibroblasts, creating a barrier-impeding migration of recipient T cells into the transplanted graft. Interestingly, the IDO expressing skin substitute also demonstrated anti-fibrotic activity by increasing MMP-1 expression in the host fibroblasts. In yet another animal model, the same group demonstrated that the IDO-expressing skin substitute accelerated wound closure and increased neovascularization in the wounded site compared to the control non IDO-expressing bio-construct [34]. This is in accordance with several lines of evidence suggesting that depleting one or more inflammatory cell types can actually have a positive outcome on the wound closure [35], [36]. Taken together, these data strongly suggest that intrinsic IDO-dependent immunosuppressive activity of fetal skin cells could not only allow their immunotolerance in an allogeneic context but also directly improve the wound healing process.

There is abundant evidences showing that growth factor-mediated cross talk between keratinocytes and fibroblasts is crucial to orchestrate the cascade of events mediating the wound healing process [37], [38]. For example, it has been largely described that keratinocytes stimulate fibroblasts to produce growth factors, which in turn will stimulate keratinocytes proliferation and migration in a double paracrine manner. This prompted us to consider the combination of both fibroblasts and keratinocytes for improvement of existing fetal fibroblasts-based therapy for cutaneous wounds. Our data showed that fibroblasts and keratinocytes secrete differential levels of numerous cytokines and growth factors including HGF, GM-CSF, IL-1α, VEGF-A and IL-8 which are well known as enhancing factors in wound healing. Among these, IL-1α is described as a primary mediator in keratinocytes-fibroblasts interactions. Keratinocytes derived IL-1α instructs fibroblasts to proliferate [39], synthesize pro-collagen type I and III [39] and produce cytokines such as HGF, GM-CSF, IL-6 and IL-8 [40]–[43]. Interestingly, in co-culture experiments, we showed that fibroblasts-keratinocytes interactions dramatically enhanced HGF, GM-CSF, and IL-8 secretion by fetal cells and also VEGF-A, although at a much lesser extent. These results strongly support that a paracrine signaling between both cell types stimulates their secretory activity and the release of multiple growth factors and cytokines [44] [45]. Comparative analysis between fetal and adult skin derived cells demonstrated similar growth factors and cytokines secretion profiles except for HGF and VEGF-A. Furthermore, in fibroblasts and keratinocytes co-culture experiments, fetal cells were found to constantly secrete higher amounts of growth factors and cytokines than adult skin derived cells.

The critical role of fibroblasts-keratinocytes interactions has been further emphasized by our *in vitro* scratch assays, showing that scratch closure was faster in case of co-culture of fetal fibroblasts and keratinocytes than in mono-cultures. This is in accordance with the striking increase in growth factors release induced by fibroblasts-keratinocytes interactions. Indeed, factors such as IL-8, HGF and GM-CSF have all been shown to stimulate keratinocytes proliferation and/or migration [46]–[49]. In addition, PDGF-AA that we found to be secreted by keratinocytes (supporting information, Figure S3) have also mitogenic and chemotactic effects on dermal fibroblasts [50], [51].

Taken together, these data show that direct interactions between fetal fibroblasts and keratinocytes strongly enhance wound healing growth factors secretion and supports the fact that a skin substitute composed of both cell types could have therapeutical benefit compared to existing fetal fibroblasts derived dressing, acting as a reservoir of growth factors.

In summary, we have successfully developed a specific protocol to isolate, expand and bank two clinical batches of keratinocytes and fibroblasts from a single fetal skin sample. Functional analyses have revealed that both fibroblasts and keratinocytes have a strong IDO-dependent-immunosuppressive activity, conferring to these cells a huge potential for wound healing improvement, and a reduced risk of allogeneic immune rejection. Moreover, our results suggest that by allowing cellular interactions between fibroblasts and keratinocytes, the combined application of both cell types should stimulate more efficiently wound healing rather than the use of one or the other single cell type. Further *in vivo* validations of these results are now required towards the development of a new well tolerated fetal derived skin substitute for cell-based therapy of skin defects.

## Supporting Information

Figure S1 **Illustration of the manufacturing process of fetal fibroblasts and keratinocytes clinical batches starting from one fetal skin sample of 18 weeks gestational age.**
(DOCX)

Figure S2 **Absence of contamination with Langerhans cell and melanocyte in fetal fibroblasts and keratinocytes clnical batches.**
(DOCX)

Figure S3 **PDGF-AA released by fetal fibroblasts or keratinocytes alone, or fetal fibroblasts and keratinocytes in co-culture.**
(DOCX)

Table S1 **Multiplex cytokine analysis of secreted factors from cell culture supernatants of fetal and adult fibroblasts and keratinocytes monocultures or co-cultures.**
(DOCX)

Video S1 **Scratch closure of keratinocytes.**
(AVI)

Video S2 **Scratch closure of fibroblasts.**
(AVI)

Video S3 **Scratch closure of keratinocytes and fibroblasts in co-culture.**
(AVI)
