# Supplementary material for: Fetal Fibroblasts and Keratinocytes with Immunosuppressive Properties for Allogeneic Cell-Based Wound Therapy
Source: PLoS One. 2013 Jul 24;8(7):e70408. doi: 10.1371/journal.pone.0070408 (PMC3722184; doi:10.1371/journal.pone.0070408)
Supplement: Figure S1 — Illustration of the manufacturing process of fetal fibroblasts and keratinocytes clinical batches starting from one fetal skin sample of 18 weeks gestational age. (DOCX) [file pone.0070408.s001.docx]

**Supplemented data, Figure S1: Illustration of the manufacturing process of fetal fibroblasts and keratinocytes clinical batches starting from one fetal skin sample of 18 weeks gestational age.** Skin sample was cut in small explants (< 0.5 cm^3^) and plated in 6-well plates at the density of 10 explants per well. Explants were cultured for 16 days in DMEM/FBS (20 %). Around some explants, a ring of keratinocytes appeared first as soon as 2-4 days of culture. Then, fibroblasts grown out of the explants and filled the wells. At D16, fibroblasts were detached from the wells by differential trypsinization, while explants and keratinocytes were kept adherent. Fibroblasts were subcultured in flask and frozen at P1 to constitute an initial cell bank (ICB). Remaining keratinocytes and explants were further cultured and medium switch from DMEM/(FBS 20 %) to CnT-07, a defined medium specific for keratinocytes amplification. At day 22, when keratinocytes reached 70-80 % confluence they were detached from the wells by trypsinization and subcultured in multiple flasks. As for fibroblasts, they were frozen at P1 to constitute an ICB. Then, one vial from both the fibroblasts and keratinocytes ICB were thawed, cells amplified for two additional passages and finally frozen to constitute two clinical batches of fetal fibroblasts and kerationocytes.
