# Supplementary material for: Fetal Fibroblasts and Keratinocytes with Immunosuppressive Properties for Allogeneic Cell-Based Wound Therapy
Source: PLoS One. 2013 Jul 24;8(7):e70408. doi: 10.1371/journal.pone.0070408 (PMC3722184; doi:10.1371/journal.pone.0070408)
Supplement: Figure S2 — Absence of contamination with Langerhans cell and melanocyte in fetal fibroblasts and keratinocytes clnical batches. (DOCX) [file pone.0070408.s002.docx]

**a**

**b**

**Supplemented data, Figure S2: Absence of contamination with Langerhans cell and melanocyte in fetal fibroblasts and keratinocytes clnical batches. a,** Cytometry histograms showing the percentage of HMB 45 (melanocytes marker) positive cells in fetal fibroblasts and keratinocytes clinical batches and in a melanoma cell line (M428) serving as positive control. **b,** Cytometry histograms showing the percentage of CD1a (Langerhans cells marker) positive cells in fetal fibroblasts and keratinocytes clinical batches and in a total epidermal cell fraction serving as positive control. Full histogramms represent test sample, solid lines represent isotypic controls.
