# Supplementary material for: Fetal Fibroblasts and Keratinocytes with Immunosuppressive Properties for Allogeneic Cell-Based Wound Therapy
Source: PLoS One. 2013 Jul 24;8(7):e70408. doi: 10.1371/journal.pone.0070408 (PMC3722184; doi:10.1371/journal.pone.0070408)
Supplement: Figure S3 — PDGF-AA released by fetal fibroblasts or keratinocytes alone, or fetal fibroblasts and keratinocytes in co-culture. (DOCX) [file pone.0070408.s003.docx]

**Supplemented data, Figure S3: PDGF-AA released by fetal fibroblasts or keratinocytes alone, or fetal fibroblasts and keratinocytes in co-culture.** The PDGF-AA release was assessed by Elisa (R&D system, Quantikine Elisa, #DAA00B, Lille, France) in culture supernatants of fetal fibroblasts, keratinocytes or fibroblasts and keratinocytes in co-culture at the ratio 1:1. For each condition, a total amount of 30 000 cells per well of 48 well plates were seeded in 450 µl of culture medium. After 48 hours incubation, supernatants were recovered, centrifuged and stored at -80 °C. CnT-07 and DMEM/SVF were used as negative control. Elisa assay was performed according to the manufacturer’s guidelines. Data shown are the means ± SD (n=3).
