# Supplementary material for: Fetal Fibroblasts and Keratinocytes with Immunosuppressive Properties for Allogeneic Cell-Based Wound Therapy
Source: PLoS One. 2013 Jul 24;8(7):e70408. doi: 10.1371/journal.pone.0070408 (PMC3722184; doi:10.1371/journal.pone.0070408)
Supplement: Table S1 — Multiplex cytokine analysis of secreted factors from cell culture supernatants of fetal and adult fibroblasts and keratinocytes monocultures or co-cultures. (DOCX) [file pone.0070408.s004.docx]

**Supplemented data, Table 1**: Multiplex cytokine analysis of secreted factors from cell culture supernatants of fetal and adult fibroblasts and keratinocytes monocultures or co-cultures. Values indicate cytokine/growth factors concentration in pg/ml.

| **Cytokines/growth factors** | **Fetal cells** | | | | **Adult cells** | | | | | | | | | | | | | | |
| --- | --- | --- | --- | --- | --- | --- | --- | --- | --- | --- | --- | --- | --- | --- | --- | --- | --- | --- | --- |
|  |  |  |  |  | **sample #1** | | | **sample #2** | | | | **sample #3** | | | | **Means for adult cells** | | | |
|  | **F** | **K** | **F/K (1 :1)** | **F** | | **K** | **F/K (1 :1)** | | **F** | **K** | **F/K (1 :1)** | | **F** | **K** | **F/K (1 :1)** | | **F** | **K** | **F/K (1 :1)** |
| **HGF** | 1 | 1 | 39 | ND | | 88 | 21 | | 3 | 70 | 25 | | 3 | 1 | 22 | | 1 | 53 | 23 |
| **GM-CSF** | 3 | ND | 562 | ND | | 110 | 250 | | ND | 150 | 214 | | ND | ND | 537 | | ND | 87 | 334 |
| **IL-1a** | ND | 157 | 76 | ND | | 125 | 32 | | ND | 121 | 31 | | ND | 131 | 38 | | ND | 126 | 34 |
| **VEGF-A** | 2770 | 1400 | 2617 | 869 | | 1935 | 2053 | | 1465 | 1989 | 2262 | | 1635 | 1700 | 2696 | | 1323 | 1875 | 2337 |
| **IL-8** | 106 | 165 | 6331 | 59 | | 870 | 5527 | | 292 | 1001 | 7027 | | 202 | 138 | 4217 | | 117 | 670 | 5590 |

F, fibroblasts mono-culture; K,keratinocytes mono-culture; F/K (1:1), fibroblasts and keratinocytes co-culture at the ratio 1:1. ND, not detectable.
